# Factors Affecting the Absorption of Subcutaneously Administered Insulin: Effect on Variability

**DOI:** 10.1155/2018/1205121

**Published:** 2018-07-04

**Authors:** A. K. J. Gradel, T. Porsgaard, J. Lykkesfeldt, T. Seested, S. Gram-Nielsen, N. R. Kristensen, H. H. F. Refsgaard

**Affiliations:** ^1^Department of Veterinary and Animal Sciences, Section of Experimental Animal Models, Faculty of Health & Medical Sciences, University of Copenhagen, Copenhagen, Denmark; ^2^Insulin Research, Global Drug Discovery, Novo Nordisk A/S, Novo Nordisk Park 1, 2760 Måløv, Denmark; ^3^Department of Histology and Imaging, Global Drug Discovery, Novo Nordisk A/S, Novo Nordisk Park 1, 2760 Måløv, Denmark; ^4^Quantitative Clinical Pharmacology, Novo Nordisk A/S, Vandtårnsvej 108, 2860 Søborg, Denmark

## Abstract

Variability in the effect of subcutaneously administered insulin represents a major challenge in insulin therapy where precise dosing is required in order to achieve targeted glucose levels. Since this variability is largely influenced by the absorption of insulin, a deeper understanding of the factors affecting the absorption of insulin from the subcutaneous tissue is necessary in order to improve glycaemic control and the long-term prognosis in people with diabetes. These factors can be related to either the insulin preparation, the injection site/patient, or the injection technique. This review highlights the factors affecting insulin absorption with special attention on the physiological factors at the injection site. In addition, it also provides a detailed description of the insulin absorption process and the various modifications to this process that have been utilized by the different insulin preparations available.

## 1. Introduction

Insulin has been widely used for blood glucose management in people with diabetes since its extraction and identification in 1921 by Banting and coworkers. However, despite significant improvements in insulin production, purification, pharmaceutical formulation, and methods of delivery, microvascular and premature macrovascular complications remain a leading cause of morbidity and mortality in these subjects [1–3].

The degree of glycaemic control influences the progression of diabetes complications in people with type 1 and type 2 diabetes [2–6]. The former depend on exogenous insulin therapy for survival, while many people with type 2 diabetes will eventually—in addition to oral hypoglycaemic agents—require insulin as a consequence of relative insulin deficiency that worsens with disease progression [7, 8].

Variability in the subcutaneous (SC) absorption and effect of insulin represents an important source of glucose variability in patients using insulin and is thus a major challenge in insulin therapy [9–11]. As the objective of insulin therapy is to mimic the normal physiological release of insulin in order to establish normoglycaemia, variability in the effect of insulin will give rise to an unpredictable therapeutic response resulting in inadequate glycaemic control and increased risk of hypoglycaemia. One important consequence of this lack of predictability is undertreatment of the disease, as many people with diabetes fear the hypoglycaemic events that are associated with overtreatment.

Although there is no formal definition of the term “glucose variability,” it is commonly understood as how blood glucose levels deviate from a mean or ideal value over time (hours and days)—a phenomenon that can also be described as “glucose fluctuation” [12]. Glucose variability is also observed as day-to-day differences in glucose values obtained at set time points, or in the 24 h blood glucose profile, and such variability goes under the term “reproducibility” or “predictability” [12].

In people using SC insulin therapy, the term “within-subject variability” usually describes differences in the blood glucose response from one injection to another in the same individual [12]. This variability contributes to glucose variability and is the sum of two components: (1) a *pharmacokinetic component*, determined by the extent and rate of absorption, distribution, and clearance of insulin and (2) a *pharmacodynamic component*, determined by insulin's metabolic effects [10]. Since the variability in insulin pharmacokinetics—which is commonly understood as variability in exposure between injections or within-subject variability—for most insulin preparations is largely determined by the absorption profile of insulin, an understanding of the factors influencing insulin absorption is necessary in terms of improving glycaemic control and the long-term prognosis in people with diabetes.

## 2. Insulin Absorption

### 2.1. Structure and Composition of Subcutaneous Tissue

The most commonly used administration route for insulin is the SC route [13].

**Figure 1:**
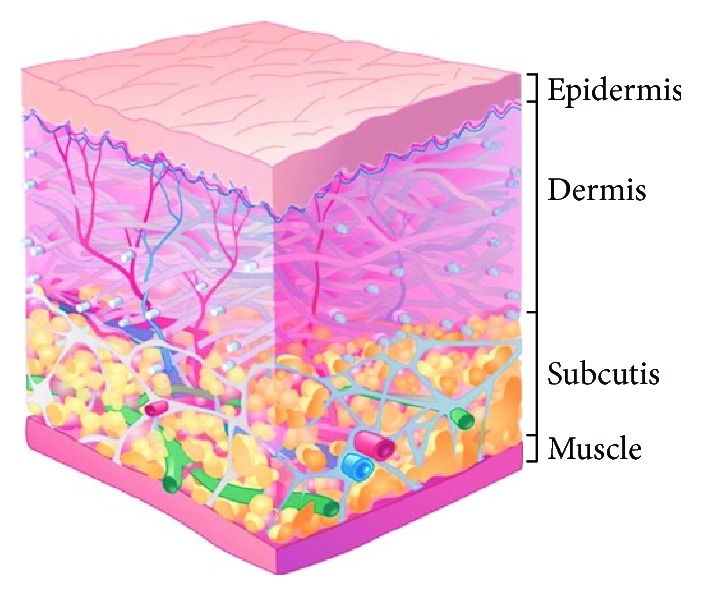
An overview of the layers of the skin and muscle. Upon injection into the subcutaneous compartment, insulin can either be absorbed by blood capillaries (red) and/or lymphatic capillaries (green). Adapted with permission from Taylor & Francis and Frost GI: Recombinant human hyaluronidase (rHuPH20): An enabling platform for subcutaneous drug and fluid administration. Expert Opin Drug Deliv (2007) 4(4):427–440. © 2007 Taylor & Francis.

The SC tissue, located between the skin (epidermis and dermis) and muscle, consists of adipose tissue separated into fat lobules by a network of connective tissue septae that are mainly composed of collagen (primarily type I, III, and V), elastin, and glycosaminoglycans (GAGs) and contain both blood and lymph vessels (Figure 1) [14–16].

Connective tissue represents the majority of the extracellular matrix (ECM). It constitutes around 10% of the SC compartment and is a physiological barrier to insulin delivery after SC administration since insulin has to travel through the connective tissue—following the path of least resistance—before entering systemic circulation [17–19]. Moreover, insulin has been reported to bind to proteins present in the extracellular matrix such as collagen that may thus act as tissue reservoirs [16]. In addition—for acylated insulin analogues—albumin binding in the SC tissue is an important mechanism in delaying absorption from the SC tissue [20].

Fibroblasts, located in the connective tissue, synthesize the components of ECM, including collagen, elastin, proteoglycans, and GAGs. The structure of SC tissue is determined mainly by collagen, while elastin provides elasticity. The negatively charged GAGs and proteoglycans attract water molecules to form the gel-like phase of the ECM and control interstitial fluid content [17]. Under normal circumstances, fibroblasts, adipocytes, and macrophages are the primary cell types residing in the connective tissue [21].

Fat lobules contain few arterioles and venules compared to the connective tissue, and they are devoid of lymphatics [17, 22, 23]. Instead, lymph capillaries are located in a plexus between dermis and subcutis where they drain into lymph vessels located in the interlobular connective tissue septae [17]. Compared to blood capillaries, endothelial cells of lymphatic capillaries lack tight junctions, allowing uptake of larger molecules [17, 24].

The interstitial fluid in the ECM derives from leakage of plasma through the blood capillaries due to pressure differences between arterioles and venules—what is not recovered by the venules is absorbed by the lymphatic system [21]. Hence, the composition of the interstitial fluid is somewhat similar to that of plasma in terms of ionic composition and pH [21]. However, it does have a considerable lower content of protein (e.g., albumin and globulin), protein-bound ions (i.e., calcium and magnesium), and free cations [25–27].

Although the dermal route may actually offer a more rapid absorption of insulin compared to the SC route, dermal injections have traditionally been more difficult to perform and may result in increased immune response activation, injection pain, and insulin leakage [28]. Microneedles may overcome some of these issues, although patients may experience more pain due to a high delivery pressure as a result of increased tissue density and decreased needle diameter [29–31], making this route less suitable for injection of larger volumes.

### 2.2. Absorption of Insulin from Subcutaneous Tissue

**Figure 2:**
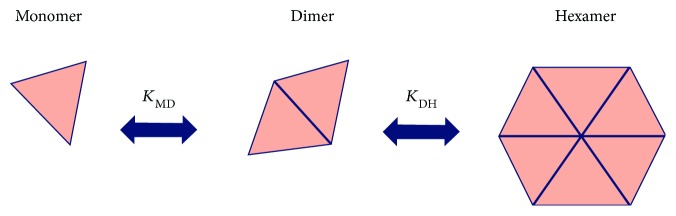
Relationship between the insulin oligomers (monomers, dimers, and hexamers) and the equilibrium constants *K*_DH_ and *K*_MD_. Adapted and printed with permission from Elsevier and Rasmussen: Insulin aspart pharmacokinetics: an assessment of its variability and underlying mechanisms. Eur J Pharm Sci (2014) 62: 65–75. © 2014 Elsevier.

Soluble human insulin consists of different oligomers in a chemical equilibrium. These include insulin monomers, dimers, and hexamers with a molecular weight of 6 kDa, 12 kDa, and 36 kDa, respectively [32]. Furthermore, certain insulin analogues also depend on the formation of dihexamers and multihexamers as a mechanism of protraction in the SC tissue [20, 33], as will be elaborated in the section on insulin types. The concentration of each insulin oligomer is determined by the equilibrium constants *K*_DH_ and *K*_MD_ between the insulin hexamers and dimers, and dimers and monomers, respectively [34] (Figure 2).

Upon injection into the SC tissue, insulin monomers and dimers are readily absorbed by blood capillaries [32]. Insulin hexamers, however, are not absorbed into the capillaries but can to some extent be absorbed by the lymphatic system due to their larger size [32, 34]. For storage purposes, excipients are usually added to the insulin formulation, and this shifts the equilibrium of insulin oligomers towards the hexamers by increasing *K*_DH_ [32, 34]. These excipients include zinc, which is needed to form insulin hexamers, and phenol and/or phenol-like substances that stabilize the hexamers and act as preservatives [32]. When injected into the SC tissue, lipophilic excipients such as phenol and meta-cresol as well as zinc disperse away from the insulin depot into the adipose tissue, which reduces *K*_DM_ allowing for the subsequent dissociation of insulin hexamers into dimers and monomers before transcapillary transport [35]. Absorption of insulin analogues generally follows a pattern similar to that of human insulin. However, due to modifications to the insulin molecule, these analogues are associated with different pharmacokinetic profiles, as will be outlined in the next section.

In order to assess pharmacokinetic variability, the factors influencing insulin absorption need to be considered. These factors are primarily related to the insulin preparation (physical-chemical factors), the injection site/patient (physiological/endogenous factors), or the injection technique.

## 3. Physical-Chemical Factors that Influence Insulin Absorption

### 3.1. Insulin Type

Since the introduction of recombinant DNA technology in the 1980s, the insulin preparations used today contain recombinant human insulin and/or analogues of human insulin as the active ingredient [7]. Based on time of onset and peak and duration of pharmacologic effect, insulin preparations can be divided into rapid-, short-, intermediate-, and long-acting insulin preparations [7, 36–40] (Table 1). The rapid- and short-acting insulin preparations are also known as prandial insulin, because they are taken at mealtime in order to cover insulin needs for glucose utilization. The intermediate- and long-acting insulin preparations are often referred to as basal insulin. They are typically only administered once or twice daily and serve to control glucose production. A basal and prandial/postprandial release of insulin to the blood stream—either from the pancreas or by means of exogenous administration—is essential in order to achieve normoglycaemia 24 hours/day. Insulin therapy should thus ideally mimic the physiological release rate of insulin as closely as possible. Moreover, some insulin products are available as mixtures in biphasic premixed or soluble formulations that provide both prandial and basal coverage [7, 41] (Table 1).

In order to elicit a metabolic effect in insulin-sensitive tissues after SC administration, insulin needs to travel through the ECM and enter the systemic circulation. As previously mentioned, the route of absorption depends on the insulin oligomer of interest: insulin monomers and dimers are readily absorbed by blood capillaries, whereas insulin hexamers are absorbed into the lymph [32, 34]. Furthermore, the oligomeric equilibrium also determines the rate of insulin absorption, since an inverse relationship exists between the association state/overall size of insulin and the fractional disappearance rate from the injection site [64]. Hence, the rate of insulin absorption is fastest for monomers followed by dimers and hexamers, respectively [35, 64]. This has been exploited in the genetic engineering of insulin analogues used in rapid-acting insulin preparations (insulin aspart, insulin lispro, and insulin glulisine), which have substitutions or minor alterations in the amino acid sequence relative to human insulin [65]. In the SC tissue—in the absence of zinc and phenol—these modifications give rise to a reduced self-association of insulin monomers into dimers compared to human insulin due to a lower *K*_MD_ and thus yield a larger fraction of insulin monomers in the SC tissue [32]. The result is a more rapid absorption from the SC tissue with faster onset of action, a higher maximum plasma concentration (*C*_max_), and shorter duration of action compared to human insulin [7, 66, 67]. Not only does the rapid absorption profile associated with rapid-acting insulin preparations allow for a more effective correction of incidental hyperglycaemia but also the preparations can be injected at a shorter time prior to meal intake, increasing the flexibility of their use [68]. Foremost, the shorter duration of action associated with these preparations reduces the need for a snack between meals in order to counteract intermeal hyperinsulinemia [69, 70]. Novel approaches in achieving even faster absorption of insulin upon SC administration include the addition of excipients such as niacinamide and L-arginine to the insulin aspart formulation [71] and citrate and treprostinil [72–75] or BioChaperone to the insulin lispro formulation [76] and coformulation of insulin with hyaluronidase [77, 78].

In contrast to rapid-acting insulin preparations, intermediate- and long-acting insulin preparations exhibit a delayed absorption profile compared to human insulin. Insulin suspensions were the only basal insulin formulations available before the introduction of insulin analogues. The neutral protamine Hagedorn (NPH) insulin preparation contains human insulin as the active ingredient. It is associated with intermediate onset of action due to the addition of the basic protein protamine to the insulin formulation that prolongs its pharmacokinetic profile [34, 79]. The crystals in the NPH suspension are formed by mixing human insulin, protamine, zinc, and phenolic substances. Whereas soluble insulin diffuses in the SC tissue, insulin crystals will remain near the injection site. Before dissociation of hexamers into dimers and monomers, these crystalline structures need to dissolve, and this process not only prolongs the absorption phase of NPH insulin but also contributes to pharmacokinetic variability between injections [10, 18, 80, 81]. In contrast to newer insulin products that are available in homogenous solutions, NPH insulin is a suspension and requires shaking before use. Consequently, inadequate resuspension represents a significant source of pharmacokinetic variability associated with NPH insulin [82]. Furthermore, the shape and size of the crystals vary between injections, and these crystals appear to be quite sensitive to changes in subcutaneous blood flow (SBF) [18]. Finally, compared to insulin analogues used in intermediate- or long-acting insulin preparations, NPH insulin is associated with peaks in exposure, which increases the risk of nocturnal hypoglycaemia when administered in the evening [83]. The result is an insulin with a pharmacokinetic profile that poorly mimics the peak-less basal release of insulin [81]. NPH insulin is also available in a biphasic mixture with short- or rapid-acting insulin preparations [7] (Table 1).

Compared to native human insulin, the insulin glargine analogue contains one modification in the amino acid sequence at position 21^A^, where asparagine has been replaced by glycine, and two arginines at the end of the B chain that are remnants from the conversion of proinsulin to insulin [84, 85]. Whereas human insulin has an isoelectric point of pH 5.4, insulin glargine has an isoelectric point of pH 7, which renders the insulin molecule soluble in acidic solution (pH 4) [84]. Upon SC injection, the acidic solution is neutralised leading to formation of microprecipitates from which small amounts of insulin glargine are continuously released to the circulation [55]. The acid pH of the insulin glargine formulation makes it challenging to mix with neutral formulations of other insulin preparation [55, 84, 86].

A different approach in reducing the rate of insulin absorption has been to attach a polyethylene glycol (PEG) polymer chain to the insulin molecule (PEGylation). This has been done in development of the PEGylated version of the analogue insulin lispro which has been reported to be associated with a half-life of 2–3 days in people with type 2 diabetes [87]. In comparison, the analogue insulin degludec, which will be introduced below, has a half-life of approximately 25 hours [88]. PEGylation not only slows insulin absorption from the SC tissue but also reduces the insulin clearance rate [89–91]. Since PEGylation increases the hydrodynamic size of the insulin molecule, most of the insulin likely enters the circulation through the lymphatic route [90, 92]. However, rather than slow lymphatic transport, the delayed SC absorption may be a result of slow interstitial transport of PEGylated insulin molecules [90]. While PEGylated insulins may provide improved glycaemic control and reduce the risk of hypoglycaemia compared to use of other analogues, concerns have been raised regarding liver fat accumulation and elevations in both triglycerides and liver enzymes which lead to termination of the PEGlispro clinical development programme [89, 93].

Another strategy to prolong the insulin absorption is by acylation of the insulin molecule—a modification implemented in the design of the analogues insulin detemir and insulin degludec. With insulin detemir, this results in an increased dihexamer formation and subsequent albumin binding at the injection site which leads to a delayed absorption from the SC tissue [20]. After entering the circulation as monomers, insulin detemir is bound to albumin, which further delays its distribution to peripheral tissues and reduces the insulin clearance rate [20]. Insulin degludec, on the other hand, exists in a dihexameric state in the insulin formulation in the presence of zinc and phenol. Upon SC injection, phenol diffuses away, which gives insulin degludec the ability to self-associate into multihexamers [33, 94] from which there is a slow, sustained release of insulin monomers [33]. The result is a 24 h coverage associated with daily injections of the insulin degludec preparation [33, 40]. The slow release of monomers from the multihexameric complexes is hypothesized to be the rate-limiting step in absorption of insulin degludec [33]. Like insulin detemir, insulin degludec binds to circulating albumin upon absorption [40, 89]. In general, albumin binding in the circulation, together with insulin solubility before and after SC injection, is considered to contribute to the observed reduction in the pharmacokinetic and pharmacodynamic variability of insulin detemir and insulin degludec [20, 33, 80, 95–97].

### 3.2. Concentration and Volume

**Figure 3:**
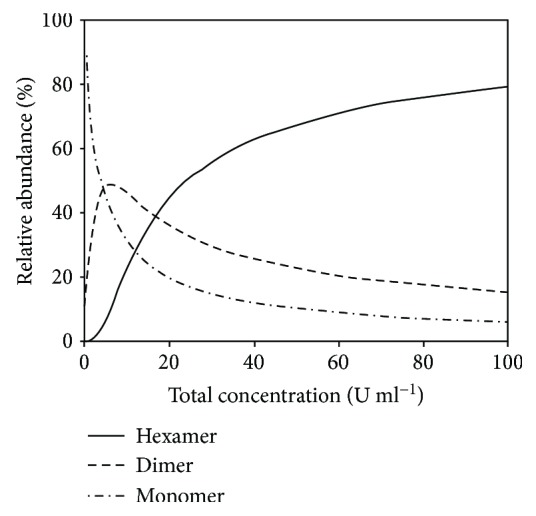
Relative abundance (%) of insulin hexamers, dimers, and monomers as a function of total concentration *C*_*τ*_. Printed with permission from Elsevier and Søeborg: Absorption kinetics of insulin after subcutaneous administration. Eur J Pharm Sci (2009) 36: 78–90. © 2009 Elsevier.

In addition to the insulin type and excipients added to the formulation [32, 34], the association state of soluble insulin is also concentration-dependent; dilution shifts the oligomeric equilibrium from hexamers towards dimers and monomers [32, 34]. Hence, the fraction of oligomers is a function of the total insulin concentration in the formulation and in the SC compartment [32] (Figure 3). Consequently, it is not surprising that an inverse relationship exists between insulin concentration and absorption rate from the injection depot [64, 98, 99].

Diffusion of insulin from the injection depot into the capillaries continuously increases the absorption rate, since a reduced concentration favours a larger fraction of monomers and dimers in the remaining depot [35]. Conversely, an increased depot concentration delays absorption and reduces the maximum plasma concentration, *C*_max_ [18]. This applies for the human insulin 500 U formulation that is associated with a reduced *C*_max_ and prolonged exposure, thus providing both prandial and basal coverage [100, 101].

The insulin concentration also affects the pharmacokinetics of insulin suspensions. For NPH insulin, the concentration of insulin crystals increases with insulin concentration with delayed SC absorption as a result [34]. Finally, the pharmacokinetics of soluble insulin that precipitates in the SC tissue is also affected by insulin concentration. The new insulin glargine 300 U formulation is associated with a prolonged pharmacokinetic and pharmacodynamic profile compared to the 100 U formulation [37, 102]. The mechanism of protraction is attributable to the reduction of the injection volume by two-thirds that results in a smaller precipitate surface area from which absorption can occur [37].

The effect of concentration does however not apply to all insulin preparations. The 200 U insulin degludec and insulin lispro formulations have both been reported to meet the bioequivalence criteria when compared to the 100 U formulations [103, 104]. For insulin degludec, it has been proposed that because the release of insulin monomers occurs at the end of each multihexameric chain, this makes the injection depot less susceptible to changes in the diffusion area associated with changes in injection volume [33, 101]. For insulin lispro, it appears that the increased zinc added to the formulation ensures bioequivalence [101, 104].

Besides a prolonged pharmacokinetic profile associated with use of some concentrated insulin preparations, their use also reduces the injection volume necessary, which in particular favours their use in highly insulin-resistant patients requiring high doses of insulin [100, 105, 106].

The effect of depot expansion can explain why higher injection volumes generally give rise to relatively slower insulin absorption [34, 98, 107]. Following SC injection, an injection depot is formed in the SC tissue that—for soluble insulin that does not precipitate in the SC tissue—diffuses and increases in volume, resulting in a concurrent dilution of insulin. The relative increase in depot volume is much faster for small compared to larger volume depots. Consequently, dilution of the depot occurs faster for small volume depots, giving rise to a relatively faster absorption [34]. In addition, a smaller depot volume will have a relatively larger surface-to-volume ratio which increases the diffusion area of the injection depot [101]. In this way, the availability of insulin pumps—which offers the advantage of injection of small volumes—or use of a dispersed injection strategy can facilitate an even more rapid absorption from the SC tissue [108, 109].

In addition to delaying insulin absorption by decreasing the surface-to-volume ratio, larger injection volumes may also exert a depressive effect on the microcirculation due to an increase in interstitial fluid pressure, thus further delaying absorption [110].

Exogenous factors influencing insulin absorption are summarized in Table 2.

## 4. Physiological Factors and Factors Related to the Injection Technique That Influence Insulin Absorption

### 4.1. Subcutaneous Blood Flow

One major contributor to the rate of insulin absorption is SBF at the injection site [9, 10]. Increased SBF results in recruitment of blood capillaries, which in turn increases the capillary exchange surface in the SC compartment. Consequently, the insulin absorption is accelerated [113, 114].

SBF is influenced by numerous factors in a complex interplay between, for example, site of injection, temperature, exercise, obesity, body position, blood pressure, use of vasodilating/vasoconstricting drugs, and smoking, many of which have been reported to influence the pharmacokinetic profile of insulin [10, 111, 113–129] (Table 3). Some of the factors will be discussed in more detail below.

### 4.2. Injection Site

The pharmacokinetic profile of insulin is affected by the injection site. Hence, the injection region and administration route (e.g., SC versus intramuscular) influence the absorption profile of insulin [98, 111, 115, 118, 122–125, 130]. This effect is probably to a large extent determined by differences in SBF between the injection sites, but differences in insulin degradation might also be a factor of importance [9, 114]. Commonly used regions for SC injection include the upper arm/deltoid, abdomen, outer thighs, and buttocks [13]. As insulin is absorbed fastest from the abdomen, slower from the arm followed by thighs and buttocks, choice of injection region may for many insulin preparations influence the metabolic response to insulin [67, 111, 115, 118, 122–125, 130, 131]. Let us look at a few examples on how different injection regions influence the pharmacokinetic profile of insulin. Injection of human insulin and insulin lispro into the abdominal region has been reported to result in greater and earlier *C*_max_ compared to the deltoid and thigh region in healthy subjects in a study by Braak et al. [125]. The difference was most evident with human insulin where the time to maximum plasma insulin concentration (*T*_max_) using abdominal injections was less than half of those achieved with the two other injection sites. *C*_max_ was also significantly lower upon injection into the thigh and deltoid compared to abdominal injection (reflected by a 32% and 42% reduction, resp.). The total insulin exposure (area under the curve; AUC_0–∞_), however, did not differ significantly between the injection sites. Also in people with diabetes, *C*_max_ has been reported to be 28% higher and occur more than twice as fast upon injection of human insulin into the abdomen compared to the thigh [122]. Moreover, different insulin absorption rates within the same region have also been reported in people with type 1 diabetes. Here, the insulin absorption was observed to occur faster after abdominal injection above compared to injections made below or lateral to the umbilicus [124]. Hence, random rotation between and within injection regions should be avoided since it likely represents a significant source of pharmacokinetic variability between injections [124, 131].

Knowledge about regional differences in insulin absorption rates helps clinicians and people with diabetes adjust insulin therapy according to specific conditions. Since a faster absorption from the abdomen results in a higher and faster onset of *C*_max_ compared to insulin injected into the thigh and vice versa [67, 122, 125, 132], the abdominal region is often the preferred site for the administration of prandial insulin as it more effectively reduces postprandial hyperglycaemia compared to, for example, the thigh [130]. In addition, it also allows for more flexible administration according to meal times. In contrast, when administrating basal insulin, it is advisable to inject into the thigh region or buttocks in order to prolong the absorption rate and reduce the injection frequency. However, the sensitivity to injection regions may not be universal. The absorption of, for example, insulin glargine and insulin degludec, has been reported not to be significantly influenced by injection region in healthy subjects, which can likely be explained by the steady state achieved with the use of these long-acting insulin preparations, as this renders their pharmacokinetic profile less susceptible to changes in absorption rate [84, 133].

Exercise and increasing skin temperatures both influence insulin absorption [99, 117, 118, 120, 126]. Heating the injection site to 40°C prior to and 60 minutes after administration of insulin aspart has been reported to reduce *T*_max_ by 42% in persons with type 1 diabetes [120]. Similarly, exercise increases the absorption rate [118, 126]. In addition, the regional differences in insulin absorption rate are likely maintained during exercise, that is, insulin is still absorbed faster from the abdominal region compared to the thigh as reported for human insulin [118]. When taking the effect of temperature and exercise into account, it is advisable to reduce the insulin dose and perhaps inject into the slower-absorbing regions under these conditions in order to reduce the risk of iatrogenic hypoglycaemia. In contrast, in order to achieve a rapid decrease in blood glucose levels, for example, in case of high postprandial hyperglycaemia, some reports suggest to inject into the abdomen and massage or heat the injection site in order to achieve the most rapid glycaemic response [116, 120, 134].

#### 4.2.1. SC versus Intramuscular Injections

For purely SC administrations, the depth of the injection does not seem to be of major importance for the insulin absorption rate [124]. Due to increased blood flow, the intramuscular administration route is associated with a more rapid insulin absorption [111, 126]. In people with type 1 diabetes, the time until 50% of the insulin has been absorbed (T_50%_) occurs twice as fast after intramuscular compared to SC injections into the thigh, and this difference is even more evident during light physical activity [126]. Accidental intramuscular injection may thus represent a major source of pharmacokinetic variability between injections, especially in lean people with type 1 diabetes [9, 141, 155]. Intramuscular administration should therefore be avoided but may be taken into use under certain circumstances (e.g., in case of ketoacidosis or dehydration) [86, 141].

#### 4.2.2. Obesity

Obesity has been reported to be associated with delayed insulin absorption [99, 114, 129, 135]. This may in part be attributed to decreased SBF as a result of an overall decrease in capillary density in the SC tissue [114, 127, 135, 136, 156]. Increased skinfold thickness has also been associated with increased steady-state insulin depot size in persons with diabetes during continuous subcutaneous insulin infusion (CSII) [157], likely as a result of delayed absorption. The considerable difference in skinfold thickness may partly explain the large between-subject variability in insulin depot size, SBF, and insulin absorption among people with diabetes [135, 157]. Despite the delayed absorption, the regional differences in insulin absorption rate appear to be maintained with obesity [114].

Dermal and SC thickness in the abdomen and thigh has been reported to be influenced by BMI, gender, and age in children and adults with diabetes in a study by Derraik et al. [158]. In this study, there was an age-dependent increase in dermal and SC thickness in children. In adults, however, aging was in contrast associated with decreased dermal and SC thickness. As expected, dermal and SC tissue thickness increased with BMI in both children and adults. Furthermore, men had an increased dermal thickness compared to women whereas women had a 19% and 80% thicker SC layer in the abdomen and thigh, respectively [158]. Similar differences in SC thickness between genders have also been reported in a study including healthy subjects where women exhibited a 30% and 95% increase in SC thickness in the abdomen and thigh, respectively, compared to men [159]. In addition to gender-related differences in anthropometry, the thickness of the SC tissue also varies within subjects from one region to another, for example, reflected by increased thickness in the abdominal region compared to the thigh [158]. Thus, differences in dermal and SC thickness represent sources of variation if not appropriately taken into account. The abovementioned observations underline the importance of choosing the appropriate needle size when administrating insulin. However, in both children and adults, even the shortest needles (4 mm) reliably transverse the skin [141]. Use of the shortest needles available is therefore recommended (the 4 mm pen and 6 mm syringe needle) as their use minimize the risk of intramuscular injection which occurs more frequently with longer needles, in slim and young patients, males, and those who use limbs rather than truncal sites for insulin injection [141] (Table 4). The importance of a correct injection technique will be discussed next.

### 4.3. Injection Technique

A proper injection technique is a prerequisite in achieving optimal glycaemic control. Therapeutic education of health care professionals and people with diabetes according to certain guidelines is therefore of uttermost importance. The international recommendations on insulin delivery provide such a guide, and the recommendations are scored according to the strength and the degree of scientific support [141]. Some of the factors related to the injection technique that can influence insulin pharmacokinetics are listed in Table 4 along with the recommendations on how to reduce the impact of these factors on the pharmacokinetic variability between injections. These factors include the importance of choosing the appropriate needle size in order to reduce the risk of intramuscular injection, how to adequately mix insulin suspensions before use, and how to avoid leakage of insulin associated with pen withdrawal or injection of larger volumes. The guidelines also provide recommendations on how to avoid lipodystrophy—a fat tissue disorder that will be reviewed more in detail later—by correct injection site rotation within injection regions and avoiding reuse of needles. Strategies for reducing pain and anxiety in persons with diabetes and recommendations on use of continuous SC insulin infusion are also included. Finally, some of the areas that should be addressed further in order for the guidelines to be improved are also highlighted. These include injections during pregnancy, injections using the newer analogues or GLP-1 receptor agonists, and injections in special populations (e.g., babies and the very elderly) or under special conditions (e.g., SC oedema). For more information on injection technique and insulin delivery, please refer to the guidelines on insulin delivery recommendations [141].

Use of jet injection, where insulin is administered at high velocity across the skin, instead of conventional pen administration has been shown to accelerate absorption of insulin aspart in healthy subjects and persons with diabetes [162, 163]. The jet injection results in the depot being dispersed in a spray-like manner in the SC tissue, thus increasing the surface-to-volume ratio of the injection depot that is likely the mechanism behind the accelerated absorption [163]. Elsemiek et al. observed a 40% shorter *T*_max_ and a significantly higher *C*_max_ with jet compared to pen administration in healthy subjects [162]. Moreover, use of jet injection has also been reported to be beneficial in terms of diminishing the obesity-associated delay in insulin absorption compared to pen injections [129, 164]. So far, the use of jet injectors in the diabetic community is limited. In addition to greater expenses, the use of jet injection devices requires proper training since inaccurate use increases the risk of incorrect dosing and mild skin trauma such as bleeding and bruising [164, 165].

### 4.4. Local Degradation at the Injection Site

Little is known about the fate of the insulin that does not reach the systemic circulation after injection into the SC tissue. Insulin degradation presumably takes place at the injection site, but the reported extent of degradation varies considerably between the few, mostly older studies that have been conducted so far [18, 111, 142–148]. Nevertheless, insulin bioavailability varies for different types of insulin preparations indicating that some degradation occurs in the SC tissue. Insulin bioavailability has, for example, been estimated to be similar for human insulin and analogues in rapid-acting insulin preparations, whereas it is lowest for insulin suspensions and biphasic insulin mixtures, decreasing with the crystal-to-soluble ratio and with increasing concentrations [18, 125]. This may partly be explained by activation of the local immune response by insulin crystals: whereas degradation of soluble insulin preparations is assumed to occur enzymatically, invading macrophages are believed to play a major role in the degradation of insulin crystals [18]. Consequently, besides affecting insulin absorption in general, SBF also influences bioavailability of crystalline insulin, specifically. This is probably due to a high sensitivity of crystalline insulin to blood flow changes, since higher SBF increases the dissolution of crystals and consequently decreases the time available for macrophage degradation [18].

Blood-derived proteases may to some degree be present in the SC tissue [17]. However, cells of the ECM can also secrete proteases, and the secretion may be stimulated by a local inflammation induced by needle penetration. Fibroblasts are most likely the source of these proteases, and their activity is regulated by a variety of cytokines [21]. Accordingly, administrations of protease inhibitors have been associated with increased insulin bioavailability [166]. Addition of the protease inhibitor aprotinin to the insulin formulation has been shown to accelerate absorption of human insulin in healthy subjects, reflected by 27% increase and earlier onset of *C*_max_. However, these results may also partly be the result of a local increase in SBF induced by aprotinin [167]. Nevertheless, due to the risk of adverse effects and insufficient data, the use of protease inhibitors in combination with insulin has so far not been approved [166].

### 4.5. Lipodystrophy

Repeated injections of insulin into the same skin area can induce lipodystrophic changes in the SC tissue [160, 161]. Lipodystrophy comprises both lipoatrophy and lipohypertrophy, and its prevalence is highest in children and young patients with type 1 diabetes [168, 169]. Lipoatrophy is believed to be caused by immunological factors, and—as with insulin antibodies—its prevalence has been significantly reduced since the availability of more purified insulin preparations and the introduction of recombinant human insulin and insulin analogues [169]. Hence, we will only review the clinical impact of lipohypertrophy in this section.

Lipohypertrophy is believed to be a nonimmunological side effect caused by the anabolic potential of insulin, occurring irrespective of administration route [7, 169, 170]. In a recent study, the prevalence of lipohypertrophy was reported to be as high as 76% and 56% in people with type 1 and type 2 diabetes, respectively [160]. Although lipohypertrophy is not believed to be caused by immunological factors, insulin antibody titres have been reported to correlate with the degree of lipoatrophy and lipohypertrophy in young people with type 1 diabetes [171]. However, a direct role of insulin antibodies in the pathogenesis of lipohypertrophy has still not been established [170].

Compared to normal SC tissue, the SC tissue in lipohypertrophied areas is more fibrous and has a poorer blood supply. Consequently, lipohypertrophy is associated with delayed insulin absorption, reduced bioavailability (potentially due to a higher degree of local degradation), and increased pharmacokinetic variability between injections [137, 138, 168]. One study conducted in people with type 1 diabetes showed that compared to injection of insulin aspart into normal abdominal tissue, injection into lipohypertrophied tissue resulted in a 25% and 22% decrease in insulin *C*_max_ and 4-hour insulin exposure (AUC_0–4h_), respectively [138]. Similar results have been reported for NPH injected into the thigh region and human insulin injected into abdomen, thigh, or deltoid [139, 140]. Repeated injection of insulin lispro into lipohypertrophic areas has been reported to result in increased variability between injections in certain pharmacokinetic and pharmacodynamic parameters in patients with type 1 diabetes [137]. Here, the coefficients of variation (CVs) for AUC_0–4h_ and *C*_max_ were as high as 52% and 55% upon injection into lipohypertrophic areas compared to 11% and 15% upon injection into normal SC tissue. Consequently, the variability in 4 h glucose exposure (AUC_GIR0–4h_) was also higher with injection into lipohypertrophic areas reflected by CVs of 57% compared to 23% in normal tissue. Others have also reported lipohypertrophy to be associated with higher prevalence of hypoglycaemia and increased glucose variability (blood glucose readings above or below 13.9 and 3.3 mM, respectively, at least three times a week) [160]. Thus, besides being perceived as an aesthetical problem, lipohypertrophy can increase pharmacokinetic variability between injections if patients repeatedly inject into these areas or rotate between lipodystrophy-affected and unaffected injection sites.

Reported risk factors for development of lipodystrophy include BMI, injection technique, number of injections per day, duration of treatment, size of the area usually used for injection, and frequency of changing injection sites and needles [160, 161, 169]. Unfortunately, many patients prefer to inject insulin into lipodystrophic tissue, since pain sensation is lower in these areas, although it worsens the condition [169]. While rotation of injection sites is highly preventive in the development of lipodystrophy [160, 161, 168], this procedure will increase the pharmacokinetic variability between injections, as mentioned earlier [131]. A compromise is therefore recommended consisting of systematic rotation within one region in order to both prevent development of lipodystrophy while simultaneously reducing the pharmacokinetic variability associated with random injection into different regions [86, 141] (Table 4). Consequently, although reducing the intraregional variability in insulin absorption due to a reduced number of injections, the use of insulin pumps may increase the incidence of lipodystrophy due to reduced capacity to rotate injection sites [170].

### 4.6. Other Factors

People with diabetes are at increased risk of developing secondary comorbidities and complications. This can alter the pharmacokinetic profile of insulin, for example, insulin absorption in patients with severe oedema, which may significantly delay insulin absorption [153]. In addition, factors such as age, gender, and anthropometry may influence insulin pharmacokinetics due to differences in volume of distribution, insulin degradation, and clearance between patients. However, the abovementioned factors likely only give rise to between-subject variability and thus insulin doses and injection strategies can be adjusted according to these factors in people requiring insulin therapy.

## 5. Discussion and Future Perspectives

Many studies that have assessed variability in insulin absorption and action so far have focused on variability between injections associated with use of different insulin preparations [80, 81, 96, 97, 103, 111, 137, 172–179]. Even under strictly controlled conditions, identical doses of all insulin preparations to some degree elicit different pharmacokinetic and pharmacodynamic responses between injections in the same patient. Several studies have investigated how physiological factors affect the absorption of insulin that naturally gives rise to pharmacokinetic variability if not taken into account; that is, if patients randomly rotate between injection sites, different absorption rates and metabolic effects may be observed from one injection site to the next. However, not many studies have assessed within-subject pharmacokinetic variability associated with, for example, repeated SC injection into tissue from obese subjects, dehydrated versus hydrated tissue, different injection regions, and tissue from patients with comorbidities, to quantify the variability associated with these variables per se. Information from such studies could help clinicians and people with diabetes adjust regimens and dosing strategies according to these factors in order to achieve the best glycaemic result.

Moreover, an area that has received even less attention in terms of assessing variability in insulin absorption is the SC microenvironment. Thus, there is a need to characterize what factors influence both distribution and kinetics of the injection depot in the SC tissue in vivo. These factors could be related to differences in the composition of interstitial fluid (e.g., ionic content, pH, and charge), the architecture of the SC tissue (e.g., collagen and elastin content), and/or proteins and cell types/density present—and these components may well vary between regions and within region over time. Furthermore, there is a need to investigate how different disease states (e.g., obesity, hypertension, and oedema) affect the SC microenvironment. This information is both of clinical relevance and may also lead to new insulin analogue candidates that may be more resistant to changes in the SC environment at the site of injection.

As previously mentioned, insulin therapy should ideally mimic the physiological release of endogenous insulin. Any imbalance between the insulin concentration profile and physiological insulin demands thus represents a source of glucose variability in people using SC insulin therapy. Consequently, reducing the glucose variability associated with SC injections of insulin also goes beyond achieving reproducible pharmacokinetic profiles, making establishment of glycaemic control in people with diabetes even more challenging. In addition to adjusting the insulin dose under certain circumstances as discussed previously (e.g., during exercise), insulin delivery systems that deliver insulin according to metabolic needs, that is, insulin pumps combined with glucose sensors that adjust the insulin dose according to blood glucose levels, likely represent an important future step in reducing glucose variability in people using SC insulin therapy.

## 6. Conclusion

Currently, all people with type 1 diabetes and many with type 2 diabetes require insulin therapy in order to achieve glycaemic control. However, variability in insulin absorption represents an important source of glucose variability in these subjects.

When assessing variability associated with the absorption of insulin, several factors need to be considered. These factors relate to the insulin preparation, the injection technique, and the individual. Education and correct instruction of clinicians and patients according to available information about such factors are essential in order to achieve the best glycaemic result, improve the long-term prognosis, and increase quality of life in people with diabetes that use insulin. However, information regarding factors driving this variability is lacking—in particular factors related to the patient and the injection site. Thus, further studies assessing endogenous factors and their contribution to the insulin absorption process are necessary in order to further improve insulin therapy.

## Figures and Tables

**Table 1:** An overview of the insulin categories, types, and available concentrations.

Category	Type	Insulin molecule	Product name	Units/millilitre and manufacturer
Prandial	Rapid-acting	Insulin aspart	Fiasp® NovoLog®/NovoRapid®	100 U (Novo Nordisk) [39] 100 U (Novo Nordisk) [42, 43]
		Insulin lispro	Humalog®	100 U, 200 U (Eli Lilly) [44, 45]
		Insulin glulisine	Apidra®	100 U (Sanofi-Aventis) [46]
	Short-acting	Human insulin	Novolin® R/Actrapid® Humulin® R U-100 Humulin R U-500	40 U, 100 U (Novo Nordisk) [47, 48] 100 U (Eli Lilly) [49] 500 U (Eli Lilly) [50]

Basal	Intermediate-acting	NPH insulin	Novolin N/Insulatard® Humulin N	40 U, 100 U (Novo Nordisk) [51, 52] 100 U (Eli Lilly) [53]
		Insulin detemir	Levemir®	100 U (Novo Nordisk) [54]
	Long-acting	Insulin glargine	Lantus® Toujeo®	100 U, (Sanofi-Aventis) [55] 300 U (Sanofi-Aventis) [37]
		Insulin degludec	Tresiba®	100 U, 200 U (Novo Nordisk) [40]

Insulin mixtures/combinations	Intermediate-acting and rapid/short-acting	NPH/human insulin	Novolin 70/30/ Mixtard® 30/40/50 Humulin 70/30	Ratio 70/30, 60/40, and 50/50 40 U, 100 U (Novo Nordisk) [56, 57] ratio 70/30100 U (Eli Lilly) [58]
		Insulin aspart protamine/aspart	NovoLog® Mix 70/30+ 50/50/ NovoMix® 30/50/70	Ratio 70/30, 50/50, and 30/70 100 U (Novo Nordisk) [59–61]
		Insulin lispro protamine/lispro	Humalog Mix 75/25+ 50/50	Ratio 75/25 and 50/50 100 U (Eli Lilly) [62, 63]
	Long-acting and rapid-acting	Insulin degludec/insulin aspart	Ryzodeg®	Ratio 70/30 100 U (Novo Nordisk) [41]

**Table 2:** Factors related to the insulin preparation and their effect on insulin pharmacokinetics.

Factor	Effect on insulin pharmacokinetics
Physical status (i) Soluble insulin (ii) Insulin suspensions (iii) Biphasic insulin mixtures	Although insulin glargine molecule is soluble in formulation, the reduced solubility at neutral pH results in the formation of microprecipitates upon SC injection with delayed absorption as a result [84]. Adding protamine to the insulin formulation results in formation of insulin crystals in the formulation which are injected into the SC tissue, thus prolonging the pharmacokinetic profile of NPH insulin (insulin suspension) [34, 79]. Suspensions and biphasic insulin mixtures are often associated with a larger pharmacokinetic variability between injection compared to insulin preparations in homogenous solution [10, 18, 80–82, 111]
Concentration	There exists an inverse relationship between insulin concentration and the insulin absorption of soluble insulin from the SC tissue, reflected by a delayed absorption with increasing insulin concentration [34, 64, 98, 99]. The effect of concentration on insulin pharmacokinetics does not apply to all soluble insulin preparations, for example, the 200 U insulin degludec and insulin lispro [103, 104]. The pharmacokinetics of insulin suspensions is also affected by insulin concentration. For NPH insulin, the concentration of insulin crystals increases with insulin concentration with delayed SC absorption as a result [34]. Finally, for soluble insulin that precipitates in SC tissue, that is, the 300 U insulin glargine formulation, increasing the concentrations will result in decreased depot surface area from which dissolution and absorption can occur resulting in delayed absorption from the SC tissue [37, 102]
Injection volume	Soluble insulin that does not precipitate in the SC tissue will diffuse and increase in volume upon SC injection, resulting in depot dilution. The relative increase in depot volume and consequently depot dilution occurs faster for small- compared to large-volume depots [34]. Smaller depots will also have a relatively larger surface-to-volume ratio that increases the diffusion area of the injection depot [101]. The result is a relatively faster absorption with smaller injection volumes [34, 98, 107]
Size	Decreased molecular size, such as the formation of insulin monomers, increases the rate of absorption [66] while increases in size of the insulin molecule by means of PEGylation [87, 90] or the self-association of insulin molecules into larger structures such as di- or multihexamers [20, 33] delay insulin absorption from the SC tissue. Furthermore, the large insulin molecules achieved by PEGylation or albumin binding of insulin reduces the insulin clearance rate and subsequently prolongs the half-life in the circulation [20, 40, 87, 89–91]
Excipients	The pharmacokinetic profile of insulin can be modified by excipients added to the formulation. Excipients such as niacinamide [112], BioChaperone [76], hyaluronidase [77, 78], citrate, and treprostinil [72–74] enhance the absorption rate of insulin by a variety of mechanisms, including effects on association state of insulin, subcutaneous blood flow, vascular permeability, insulin diffusion, or depot distribution in the SC tissue, while protamine, zinc, and phenol [32, 34] can also influence the absorption of certain insulin molecules by altering the association state of insulin

**Table 3:** Factors related to the injection site/patient that influence insulin pharmacokinetics.

Factor	Effect on insulin pharmacokinetics
Subcutaneous blood flow (SBF) at injection site	Increased SBF accelerates insulin absorption [113, 114]. SBF is influenced by several factors. Increasing temperatures [113] and exercise [128] increase SBF, whereas obesity [114, 127, 135, 136] and smoking [119] decrease SBF. SBF is also increased in the abdomen and arm/deltoid compared to the thigh and buttocks [114] and during a supine compared to sitting position [121]
Lipohypertrophy	Lipohypertrophy delays absorption, and injection into these areas increases within-subject pharmacokinetic and pharmacodynamic variability between injections [137–140] and should be avoided [141]
Skin temperature	Increasing skin temperatures accelerate insulin absorption [99, 113, 115, 117, 120]
Local degradation	Affects the bioavailability of insulin, which is lower for insulin suspensions and biphasic insulin mixtures compared to soluble insulin [111, 142–148]
Local massage	Massage of the injection site accelerates insulin absorption [115, 116, 134], likely due to an increased insulin depot surface-to-volume ratio and not increased SBF [134]
Injection site (i) Abdomen, arm/deltoid, thigh, or buttocks	Insulin is more readily absorbed from the abdomen and deltoid region compared to thigh and buttocks [111, 115, 118, 122–125, 130]. The pharmacokinetic profile of long-acting insulin preparations has been reported to be less susceptible to changes in absorption rate associated with injection site rotation [84, 133].
Administration route (i) Subcutaneous versus intramuscular	Insulin is absorbed faster after intramuscular compared to SC injections [126, 149]. Intramuscular injections should be avoided as they increase the risk of hypoglycaemia [141] but may be taken into use under certain rare circumstances (e.g., in case of ketoacidosis or dehydration) [86]
Blood glucose levels	Hypoglycaemia has been reported to have no influence [150, 151] or decrease [152] the absorption of insulin in healthy [151, 152] and diabetic subjects [150].
Diabetes related comorbidities and complications	For example, oedema has been reported to delay SC absorption [153]
Obesity	Obesity gives rise to a decreased insulin absorption rate [99, 114, 129, 135]. High variation in skinfold thickness between patients may contribute to the high pharmacokinetic variability between people with diabetes [135]
Exercise and activity level	Exercise accelerates insulin absorption [118, 126], and therefore, the insulin dose should be adjusted accordingly to reduce the risk of iatrogenic hypoglycaemia [154]
Smoking	Causes peripheral vasoconstriction and delays insulin absorption [119]
Body position	Compared to a supine position, a sitting position is associated with reduced SBF and delayed insulin absorption [121]

**Table 4:** Factors related to the injection technique that influence insulin pharmacokinetics and international recommendations on insulin delivery that aim at reducing pharmacokinetic variability between injections.

Factor	Effect on insulin pharmacokinetics	International recommendations on insulin delivery [141]
Needle size	Age and gender, for example, have significant influence on the anthropometry in people with diabetes and should therefore be taken into account when choosing needle length and dosing strategy [158] in order to reduce the risk of intramuscular injection	Use of the shortest needles is recommended (the 4 mm pen and 6 mm syringe needle). In order to decrease the risk of intramuscular injections, the 4 mm needle should be used for injection in children and young adults. Lifting of a skinfold prior to injection or injection at a 45° angle may further reduce the risk of intramuscular injection
Time before withdrawal	Rapid withdrawal may result in loss of insulin and increased pharmacokinetic variability between injections [141]	With use of insulin pens, patients should count to 10 after the plunger is fully depressed before removing the needle from the skin
Dispersion	Dispersion of the injection volume gives rise to a more rapid absorption [108]	Larger doses may be split to reduce the volume of insulin and avoid leakage
Mixing	Inadequate resuspension is a problem with insulin suspensions (e.g., NPH insulin) and contributes to pharmacokinetic variability between injections [82, 141]	It is recommended to gently roll and tip cloudy insulin until the crystals are resuspended (the solution becomes milk white)
Needle reuse	Reuse of needles increases the risk of lipodystrophy [160, 161]	Reusing insulin needles is not an optimal injection practice, and patients should be discouraged from doing so
Rotation	Rotation between injection sites reduces the prevalence of lipodystrophy [141, 160, 161], but for a number of insulin preparations, rotation also elicits different pharmacokinetic and pharmacodynamic responses [111, 115, 118, 122–125, 130]	Patients should be encouraged to avoid injecting into areas of lipohypertrophy, and injections should be rotated by injecting at least 1 cm from previous injection (i.e., within the same injection region)
